# Effect of different inner core diameters on structural strength of cannulated pedicle screws under various lumbar spine movements

**DOI:** 10.1186/s12938-017-0392-1

**Published:** 2017-08-15

**Authors:** Chia-Ming Chang, Yu-Shu Lai, Cheng-Kung Cheng

**Affiliations:** 1 Department of Biomedical Engineering, National Yang-Ming University, No. 155, Sec. 2, Linong Street, Taipei, 112 Taiwan; 2 Orthopaedic Device Research Center, National Yang-Ming University, No. 155, Sec. 2, Linong Street, Taipei, 112 Taiwan

**Keywords:** Cannulated screw, Finite element analysis, von-Mises stress, Stiffness

## Abstract

**Background:**

Currently, cannulated pedicle screws have been widely used in minimal invasive or navigation techniques. However, the stress distribution and the strength of different core diameters of cannulated screw are not clear. This study aimed to investigate the mechanical strength of cannulated screws with different inner core diameter under various lumbar spine movements using finite element analysis.

**Results:**

The results showed that the von-Mises stress of a cannulated screw was larger than that of a solid screw in all loading conditions, especially above 2 mm in cannulated core diameter. In lateral bending, extension, and flexion, the maximum von-Mises stress was found approximate to the proximal thread for all types of screws. In rotation condition, the maximum von-Mises stress was located at the middle of the screw. Additionally, the difference in stiffness of instrumented levels was not significant among four screws under the same loading condition.

**Conclusion:**

Cannulated screws could provide enough stability for the vertebral body fusion comparing to solid screws. The diameter of cannulated core is suggested not to exceed 2.0 mm.

## Background

**Fig. 1:**
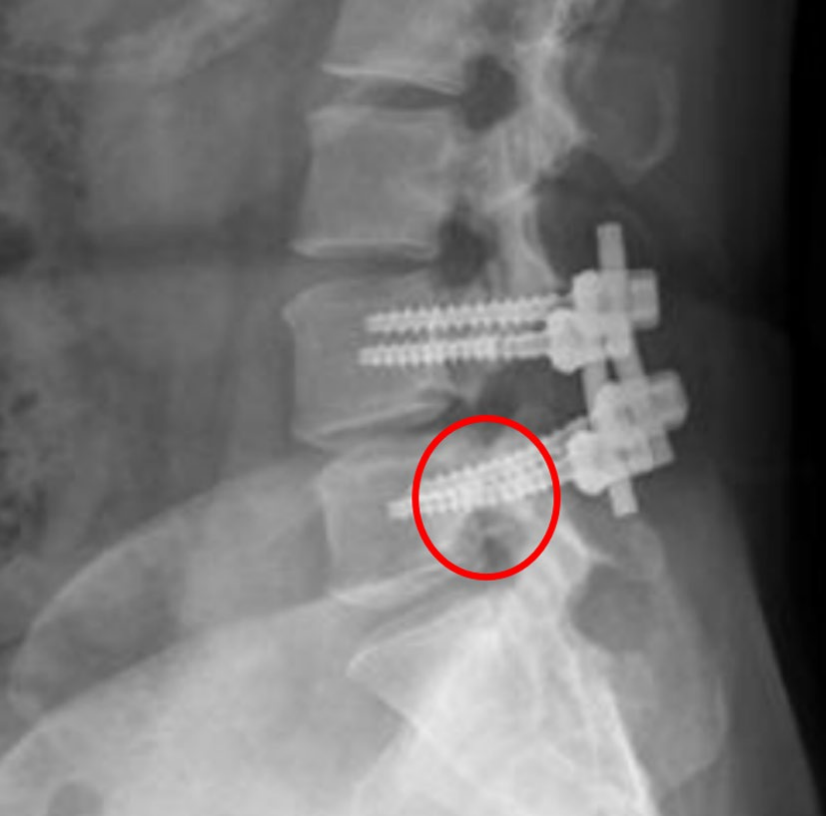
Radiographic of screw: radiographic examination showed the screw breakage (*red circle*)

Spinal fixation instrument is commonly used to improve the stability in spinal surgeries. Spinal fusion with fixation instruments has become more popular than the fusion without instruments [1, 2]. The major functions of these fixation instruments, especially pedicle screws, are enhancing fusion with bone grafts and correcting the spinal deformity,

The cannulated pedicle screw is an alternative design of traditional solid pedicle screw, which was developed to be applied in minimal invasive or navigation techniques and inserted following the guidance wire along the planned path [3]. Although 3.0 to 12.4% failure rate of a solid pedicle was reported in the literatures, [4–7], the prevalence of cannulated screw breakages remains unclear. From 2012 to 2014, we have reviewed seventy cases with cannulated pedicle screw in posterolateral lumbar fusion and found a total of eighteen screws breakage cases post-operatively (Table 1; Fig. 1). The failure rate of cannulated screws was about 18%, which seem to be larger than that of the solid screws in our experience. Of the twelve cases with cannulated screws breakage we reviewed, the breakages occurred at 4–5 months in average after receiving the posterolateral lumbar fusion surgeries (Approved by the National Yang-Ming University, YM105003E). In addition, among the eighteen broken screws, fourteen (78%) broke at either the first or the second thread counting from the screw head, and four (22%) broke at the middle of the screw.

**Table 1:** Summary information of breakage cannulated pedicle screw

Variables	Value
Age (years old)	59.1 ± 10.3
Gender (male: female)	7: 11
Failure time (months)	4.7 ± 2.6
Failure location (neck: shaft)	14: 4

Chen et al. [8] evaluated the pedicle screw breakage by conducting a retrieval analysis and found that the screw breakages were near the junction between screw head and shaft of the second thread in 75% of the patients. They also indicated that the highest stress was concentrated at the bone/screw interface. Although there was no discussion about the risk of cannulate screw breakage in spinal fixation, Glasgow et al. [9] raised concerns about the increased risk of screw breakage in metatarsal distal fixation using cannulated screws. Generally, there are two primary failure mechanisms leading to a screw breakage. One is due to the excessive torque to overcome the resistance during screw inserting into the pedicle as the consequences of small pilot hole or untapping hole in the cortical bone. High shear stress may be developed in the cross section of the screw when it is inserted under a significant resistance torque [10]. The other failure mechanism is bending of the screw as a cantilever with partial shaft location lodged in bone and a perpendicular load applied to the long axis of the screw.

Pedicle screw breakage is one of the most common complications in spinal fusion surgeries, especial for multi-levels fusion. Different inner diameter could influence the structure strength of the cannulate pedicle screw, and at least 1.5 mm would be necessary to work with the guidance wire. Therefore, the purpose of this study was to investigate the mechanical strength of cannulated screws with different inner core diameter under various lumbar spine movements using finite element analysis. We hypothesized that the core cannulated path may lead to stress concentrations and may become a source of crack that cause failure of screw. In addition, larger inner core diameters are associated with significant increased risk of screw breakage.

## Methods

### Finite element analysis models

**Fig. 2:**
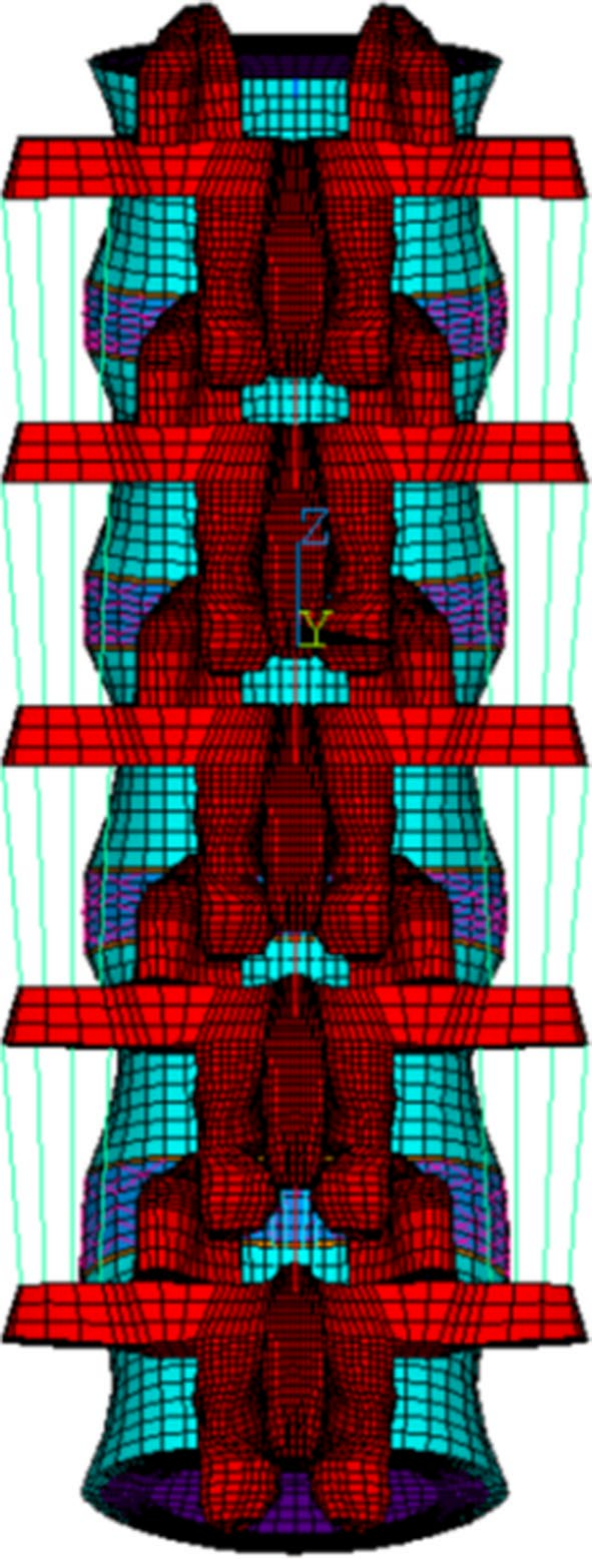
The finite element model: the FE model of the L1–L5 lumbar spine included ligaments, disc, vertebral body, and posterior element

The intact lumbar spine model (L1–L5), which had been validated by comparison with in vitro tests and successfully used for biomechanical analyses [11–13], was reconstructed. The model consisted of vertebrae, intervertebral discs, superior and inferior facet articulating surfaces, and a number of ligaments including supraspinous, interspinous, ligamentum flavum, transverse, posterior longitudinal, anterior longitudinal, and capsular. Cable elements were used to simulate ligaments and the annulus fiber of discs, which were only activated in tension. The contact behavior of the facet articulation were simulated using three-dimensional contact elements (Fig. 2). In order to analyze the failure risks in the pedicle screws, the intact finite element (FE) model of the lumbar spine was implanted with screws, rods, and bone graft elements to simulate spinal fusion. The screw and rod were made of titanium alloy (Ti6Al4V) and modeled as 8-nodes tetrahedral elements. The material properties of all components were adopted from literatures (Table 2) [14–18].

**Table 2:** The material properties specified in the finite element models [8, 12]

	Young’s modulus (MPa)	Poisson’s ratio	Cross-sectional area (mm^2^)
Bony structure			
Cortex	12,000	0.3	–
Trabecular bone	100	0.2	–
Posterior element/graft bone	3500	0.25	–
Intervertebral disc			
Nucleus pulposus	1	0.49	–
Ground substance	4.2	0.45	–
Annular fiber	175	–	0.76
Ligaments			
Anterior longitudinal ligament	7.8	–	63.7
Posterior longitudinal ligament	10	–	20
Intertransverse ligament	10	–	1.8
Ligamentum flavum	15	–	40
Interspinous ligament	10	–	40
Suprespinous ligament	8	–	30
Capsular ligament	7.5	–	30
Implants			
Bone graft	3500	0.25	–
Titanium alloy	180,000	0.28	–

The posterolateral fusion model of L3/L4, which is the most common involved level, was used to simulate changes in post-operative conditions. In the posterolateral fusion model, the bone graft elements were placed between two adjacent transverse processes to simulate perfect fusion. The average width, thickness, and length of the bone graft was 13.1, 11.3, and 41.6 mm, respectively, with a volume of 6163 mm^3^.

**Fig. 3:**
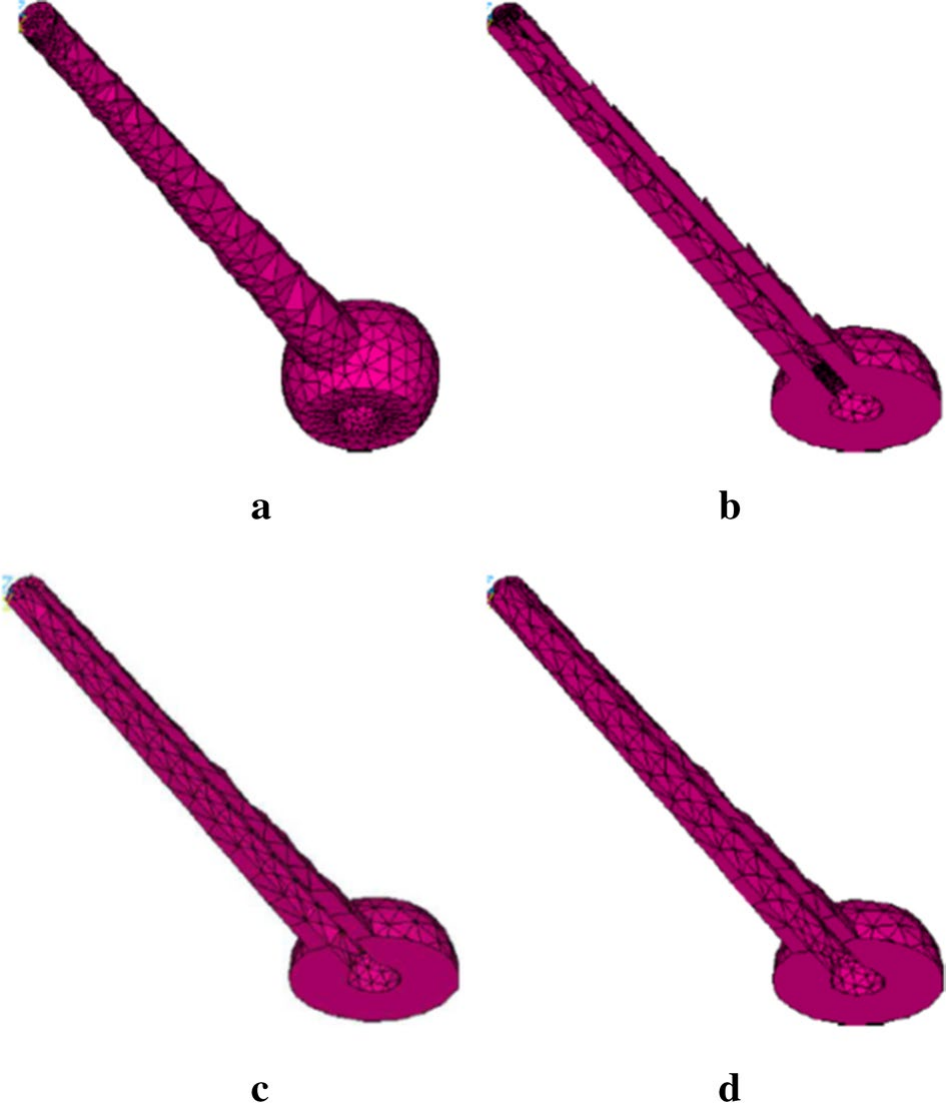
Four screw types were analyzed in this analysis: **a** a solid screw (S0); **b** a screw combining with 1.5 mm cannulated core path (S1.5); **c** a screw combining with 2.0 mm cannulated core path (S2.0); **d** a screw combining with 2.5 mm cannulated core path (S2.5)

The pedicle screw was reconstructed with an outer diameter of 6.5 mm and a length of 45.0 mm which is the most commonly used size in clinical. A total of four screws were designed in this study, including a solid screw (S0), a screw combined with a 1.5 mm cannulated core path (S1.5), a screw combined with a 2.0 mm cannulated core path (S2.0), and a screw combed with a 2.5 mm cannulated core path (S2.5; Fig. 3). The 5.5 mm rod was applied to L3/L4 posterolateral fusion. Mesh convergence test for von Mises stress under flexion loading in all of these screw models were performed. The number of elements were 2625, 2603, 2689 and 2735 for the S0, S1.5, S2.0 and S2.5 respectively.

### Boundary conditions

The FE method was used to analyze and compare the von-Mises stress performance among the four screws under physiological movements, including flexion, extension, lateral bending, and rotation. In the FE models, all degrees of freedom of the nodes at the bottom of L5 vertebral body were fixed. A pre-compressive loading of 150 N followed by moments of 10 Nm torsion, 10 Nm lateral bending, 10 Nm extension, and 10 Nm flexion were applied separately at the superior surface of the L1 using ANSYS 11.0 simulation software (ANSYS Inc., Canonsburg, PA, USA) [8, 11, 12]. The moment and axial load applied on the model represented the combined contributions in physiological responses and not to showed actually an average or peak value of each own during daily activities. The interface between the screw and bone as well as between the screw and rod were all set as bounded. The stiffness of the fused level of the four screws under different loading moment was calculated in the FE model, and the von-Mises stresses of screws were recorded.

## Results

The von-Mises stress of the screws increased with the increasing inner core diameters (Table 3). Flexion and lateral bending resulted in a much higher stress level in all screw types compared to rotation and extension. In addition, the results also indicated that the lower pedicle screw (L4) resulted in a larger stress than the upper screw (L3) under general loading conditions, except for the extension movement.

**Table 3:** The maximum von-Mises stress (MPa) on the screw of S0, S1.5, S2.0 and S2.5 due to four loading conditions

Loading		Screw							
		S0		S1.5		S2.0		S2.5	
		Left	Right	Left	Right	Left	Right	Left	Right
Rotation									
L3		4.6	8.2	6.2	11.0	5.5	9.7	6.5	11.3
L4		5.6	7.5	4.8	4.8	8.5	20.3	10.5	21.0
Lateral bending									
L3		46.7	50.6	47.7	58.4	56.7	56.7	62.2	54.7
L4		45.0	54.7	47.7	68.3	51.1	69.2	60.8	77.5
Extension									
L3		61.1		47.4		58.0		62.9	
L4		71.2		42.8		51.5		57.8	
Flexion									
L3		61.1		62.0		71.6		78.1	
L4		71.2		74.0		82.9		89.6	

**Fig. 4:**
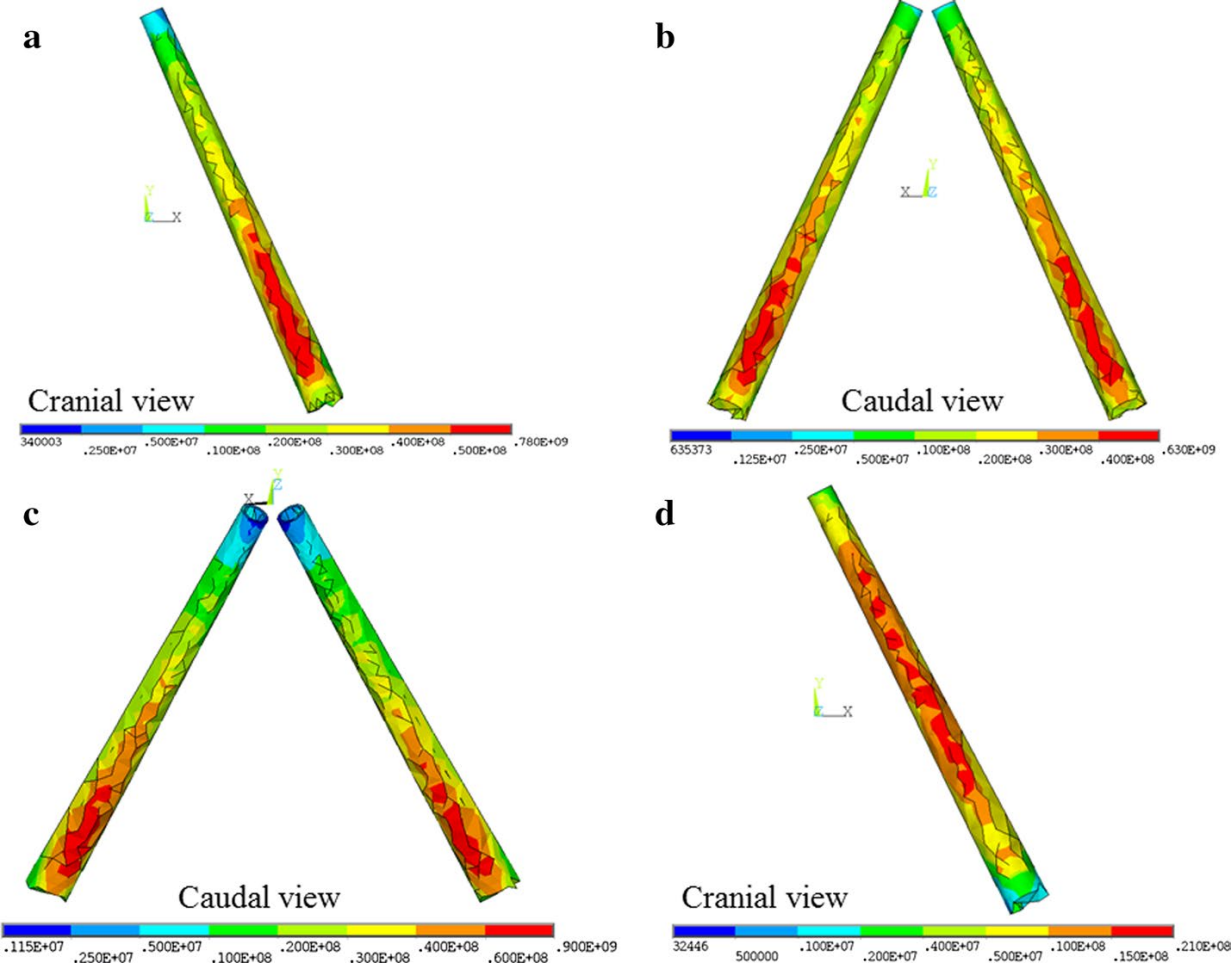
The maximum von-Mises stress (Pa) of S2.5 in L4 under following loading: **a** lateral bending, **b** extension, **c** flexion, and **d** rotation

In rotation, the maximum von-Mises stress of the S1.5, S2.0, and S2.5 increased by 87.5, 150.0, and 162.5% compared to the S0. The maximum von-Mises stress concentrated at the middle of the lower screw. In lateral bending, the maximum von-Mises stress of the S1.5, S2.0, and S2.5 increased by 23.6, 25.4, and 41.8% compared to the S0. The maximum von-Mises stress occurred at the proximal thread of the lower screw. In extension, the maximum von-Mises stress of the S1.5, S2.0, and S2.5 increased by 0, 18.4, and 28.6% compared to the S0. The maximum von-Mises stress was found at the proximal thread of the upper screw. In flexion, the maximum von-Mises stress of the S1.5, S2.0, and S2.5 increased by 4.2, 16.9, and 26.8% compared to the S0. The maximum von-Mises stress occurred at the proximal thread of the lower screw (Fig. 4).

The stiffness of different screw at the fused level under each loading condition was showed in Table 4. In the same loading condition, the difference of fused level stiffness was the least among the four screws.

**Table 4:** The stiffness (Nm/degrees) of intact spine, S0 screw, S1.5 screw, S2.0 screw, and S2.5 screw model in four loading conditions

Groups loading	Intact	S0	S1.5	S2.0	S2.5
Rotation	4.75	5.40	5.39	5.41	5.41
Lateral bending	3.05	9.46	9.48	9.38	9.48
Extension	5.40	21.02	20.96	20.69	20.38
Flexion	3.27	10.75	10.95	10.77	11.03

## Discussion

The cannulated screws have become increasingly popular in orthopedic surgeries, including foot, ankle, hip, small joint, and spine fixation over past 10 years. However, the main concern of using a cannulated screw is that its mechanical strength is lower than that of a solid screw with a similar diameter [19]. Yang et al. [20] analyzed the axial stiffness and maximum failure strength of cannulated locking screws and solid locking screws under bending moments using in vitro tests. They showed that the solid screws had higher stiffness and axial failure loads than the cannulated screws. Yang et al. also suggested that fixations with solid locking screws offered more stability compared with locking screws with regarding to axial stiffness and failure strength in unstable proximal tibial fractures. However, the difference of mechanical strength in cannulated and solid screws is not clear. Hence, the FE models were created to evaluate this difference, and the failure risks of cannulated screw with different inner core diameter were evaluated by quantifying the von-Mises stress of the screws under spinal axial rotation, lateral bending, extension, and flexion conditions.

The primary function of pedicle screw system is to stabilize the spine and share the physical loading. If the pedicle screws were broken in the early postoperative period, patients with failed constructs, which may develop progressive kyphosis subsequently, could have poor functional outcomes, progressive back pain, and may require additional surgical procedure for instrument removal [21]. The cannulated screw breakage was also reported in humerus and femoral fracture fixation [22–24].

The FE analysis indicated that the inner core diameter could affect the stress distribution of the whole screw. The maximum von-Mises stress increased as the inner core diameter increased. According to the results, the maximum von-Mises stress of the screw occurred at the caudal side of the screw during flexion, extension and laterals bending, but concentred at middle part during torsion (Fig. 4). The largest maximum von-Mises stress of all screws concentrated in flexion condition while the smallest maximum stress occurred in rotation. Compared to the S0 screw among the four loading conditions, the von-Mises stress of the S2.5 and the S2.0 screws were increased obviously, but the von-Mises stress the S1.5 screw was only increased in lateral bending and rotation. The stresses of the three cannulated screws were increased over 80% in torsion condition. Nevertheless, the loading of the screw was low in rotation, even if the von-Mises stress of the S2.5 screw increased by 160%, this value is still lower than the stress of a solid screw in flexion and extension condition. Therefore, the possibility of torsion failure type in the cannulated pedicel screw is relatively lower than the solid screw, and this result is compliance with the reduced rate of middle part screw breaking in clinical cases. The analysis showed that the maximum von-Mises stress of all groups did not reach the yield strength, but relatively short fatigue life of screw if it is under high stress amplitude. Hou et al. [25] found that the fatigue life of the commercially available titanium tibia locking screws decreased as the stress increased. In the current analyses, the stress of the S2.0 and the S2.5 screw under four loading conditions were larger than the solid pedicle screw, indicating that the fatigue life will be consequentially reduced. Therefore, before patients receive a solid fusion, the failure possibility of cannulated screws is higher than that of normal screws. Besides, Rolmann et al. [26] indicated that the spinal instrumentation was highly loaded in daily activity with good fixation. This may explain the fatigue loading and screw failure even with a solid bone-implant interface. Although the increased diameters of cannulated core caused high stress along the screw, the stiffness of the pedicle screw structure was not affected by the cannulated core (Table 4). Therefore, the cannulated screw could provide enough stability for vertebral body fusion, and the diameter of cannulated core over 2 mm should not be used.

In this study, the stress of screws was analyzed to identify the failure locations of clinical cases. Besides, the diameter of cannulated core affecting the maximum stress of pedicle screw under different loading conditions has been shown. Several limitations to this FE model analyses are noted:

1. The screw model in this study was designed according to commercial products. The diameter of the cannulated core is the only factor being discussed. Other factors that might affect the test result, such as the screw type, pitch, and thread’s shape, will be investigated in the future.
2. Single-level instrumented lumbar posterolateral fusion was simulated in this model. Multi-levels posterolateral fusions were not analyzed in this study.
3. The material properties of the vertebral body were assumed to be isotropic and homogenous.
4. The loading conditions were not truly physiological condition because this model did not account for the mechanical effects of muscle contraction.

Although the models incorporated some assumptions and limitations, the results showed a similar pattern and trend to previous studies and clinical cases.

## Conclusions

Because the diameter of a guide pin defines its stiffness, it is important for the cannulated path of the screw to accommodate as much width as possible. A wide diameter guide pin could restrict bending when it is inserted. However, this study demonstrated that cannulation width caused a raise of stress concentration at the proximal screw shaft, and it could further increase the fatigue failure risks. From the results, the diameter of the cannulated core is suggested not to exceed 2.0 mm. Even a screw with a diameter of around 1.5 mm could decrease the effects of the loss of a screw’s strength.
